# A Radiolucent Electromagnetic Tracking System for Use with Intraoperative X-ray Imaging

**DOI:** 10.3390/s21103357

**Published:** 2021-05-12

**Authors:** Kilian O’Donoghue, Herman Alexander Jaeger, Padraig Cantillon-Murphy

**Affiliations:** 1 Tyndall National Institute, Dyke Parade, T12 R5CP Cork, Ireland; alexander.jaeger@tyndall.ie (H.A.J.); p.cantillonmurphy@ucc.ie (P.C.-M.); 2 School of Engineering, University College Cork, T12 K8AF Cork, Ireland

**Keywords:** electromagnetic tracking, radiolucent devices, magnetic fields, X-ray imaging

## Abstract

In recent times, the use of electromagnetic tracking for navigation in surgery has quickly become a vital tool in minimally invasive surgery. In many procedures, electromagnetic tracking is used in tandem with X-ray technology to track a variety of tools and instruments. Most commercially available EM tracking systems can cause X-ray artifacts and attenuation due to their construction and the metals that form them. In this work, we provide a novel solution to this problem by creating a new radiolucent electromagnetic navigation system that has minimal impact on -ray imaging systems. This is a continuation of our previous work where we showed the development of the Anser open-source electromagnetic tracking system. Typical electromagnetic tracking systems operate by generating low frequency magnetic fields from coils that are located near the patient. These coils are typically made from copper, steel, and other dense radiopaque materials. In this work, we explore the use of low density aluminum to create these coils and we demonstrate that the effect on X-ray images is significantly reduced as a result of these novel changes in the materials used. The resulting field generator is shown to give at least a 60% reduction in the X-ray attenuation in comparison to our earlier designs. We verify that the system accuracy of approximately 1.5 mm RMS error is maintained with this change in design.

## 1. Introduction

In recent times, the use of electromagnetic tracking (EMT) for navigation in surgery has quickly become a vital tool in a wide range of minimally invasive surgeries [1,2,3,4,5]. The technology allows for the real-time navigation of instruments without direct line of sight [6]. EMT is an ever-expanding area of research and development with numerous new systems in development aiming to improve the performance and robustness of the technology across both commercial and academic settings [3,6,7,8,9]. One of the main areas of development in recent times is the application of machine learning algorithms to overcome some of the shortcomings of the technology [7,10,11]. Commercially available medical systems include the Aurora by Northern Digital Inc and Calypso by Varian Medical System Inc [6].

Pre-operative imaging modalities such as CT and MRI are frequently used to create the 3D visualizations typically used in conjunction with electromagnetic tracking [12]. However, the inherent registration errors and drift in the position of the patient in comparison to the pre-operative image can cause significant errors in tracking [4,13,14]. This coupled with breathing artifacts and other patient movements can limit the effectiveness of electromagnetic tracking in certain instances [9]. To counter these effects, physicians frequently utilize inter-operative imaging such as fluoroscopy for intermittent verification of the true position of instruments [3,4,5].

From this co-presence of the electromagnetic tracking hardware and the X-ray technology, interference and image artifacts are a common result [5,15,16]. EMT hardware often comprises of planar field generators that lie below the patient throughout the procedure as they can offer easier integration into the OR workspace [1,17]. For inter-operative X-ray imaging, the presence of the field generator below the patient can cause image artifacts due to the dense metals often used for the field generator boards [5,13,16] both from the copper frequently used in the windings as well as the ferromagnetic materials often used for shielding and coil core materials [18].

**Figure 1:**
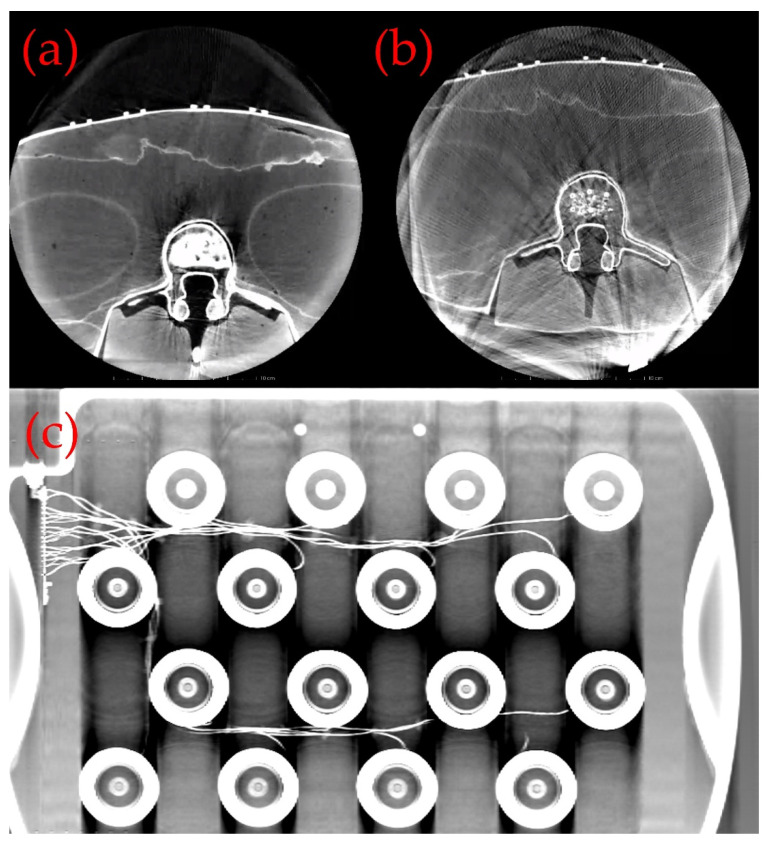
An example of the distortion created by a commercially available, planar field generator. (**a**) shows a control CT image of a spine phantom (Cirus 057A) and (**b**) shows the significant image artifacts caused by the addition of the field generator. (**c**) shows a planar X-ray image of the same device and the clear artifacts of the dense metal coils.

The impact of these distortions is significant in both CT imaging and planar radiography and has been observed in practice in many instances by our group. Figure 1 shows an example of the distortion created by a commercially available, planar field generator. Figure 1a shows a control CT image of an abdominal phantom (Cirus 057A) and Figure 1b shows the significant image artefacts caused by the addition of the field generator. Figure 1c shows a planar X-ray image of the same device and the clear artifacts of the dense metal coils.

To address this significant problem, we propose a new approach to field generator design for EMT that uses low-density, radiolucent materials for the coil design to mitigate these image distortion effects [19]. This change in material brings with it significant additional challenges that will be explored here.

## 2. Materials and Methods

### 2.1. EM Tracking

**Figure 2:**
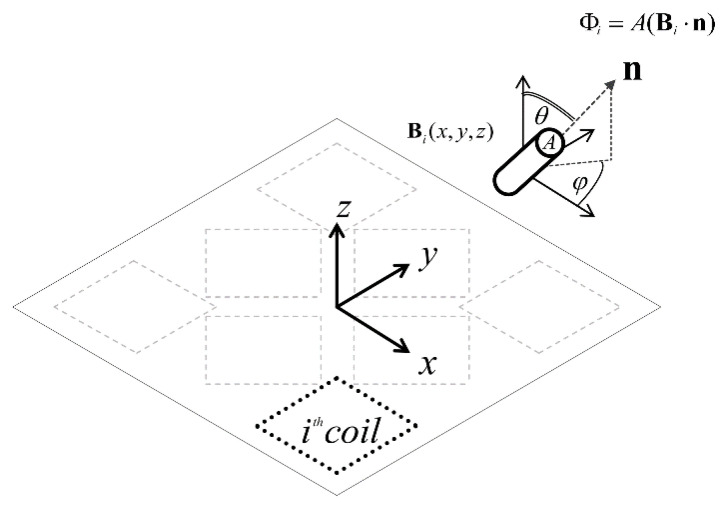
Coordinate system for the tracking algorithm. The sensor is positioned at (*x*,*y*,*z*) with an orientation denoted by *θ* and *φ*. The magnetic field resulting from the *i*th coil is indicated and the associated flux is determined using the dot product between the sensor’s directional unit vector and the magnetic field at that point [17].

AC electromagnetic tracking systems are typically based on Faraday’s law to induce a voltage in a search coil [1,17,20,21]. EM tracking generally operates with magnetic fields from multiple sources that are spatially varying functions given by:

(1) $$B_{i}(x,y,z)=\left[\begin{array}{ccc} B_{x}{}_{i} & B_{y}{}_{i} & B_{z}{}_{i} \end{array}\right]$$

where *x*, *y*, and *z* are the Cartesian coordinates of a sensor relative to the magnetic source. The flux at a given point is detected, which results in a 5 degree of freedom (DOF) equation of position and orientation as follows:

(2) $$\Phi_{i}=\;A\left[B_{x}{}_{i}\sin\theta \cos\varphi +B_{y}{}_{i}\sin\theta \sin\varphi +B_{z}{}_{i}\cos\theta \right]$$

where the orientation of the sensor in spherical coordinates is represented with the two variables *θ* and *φ,* and the sensor has a cross sectional area given by *A*. Figure 2 shows a schematic representation of this formulation. 

Once the flux has been measured, the position and orientation of the sensor can be determined by using a non-linear least squares algorithm such as the Levenberg–Marquardt (LM) method [17]. The objective function to be solved for a set of *n* magnetic sources is given by

(3) $$F(x,y,z,\theta ,\phi )=\sum_{i=1}^{n}{\left(\Phi_{meas}^{i}-\Phi_{calc}^{i}\right)}^{2}$$

which is the sum of squares of the difference between the measured flux $\Phi_{meas}^{i}$ and the flux calculated using models of the magnetic field $\Phi_{calc}^{i}$. For convergence of the algorithm, the number of magnetic sources should be greater than or equal to the degrees of freedom, i.e., *n* ≥ 5.

**Figure 3:**
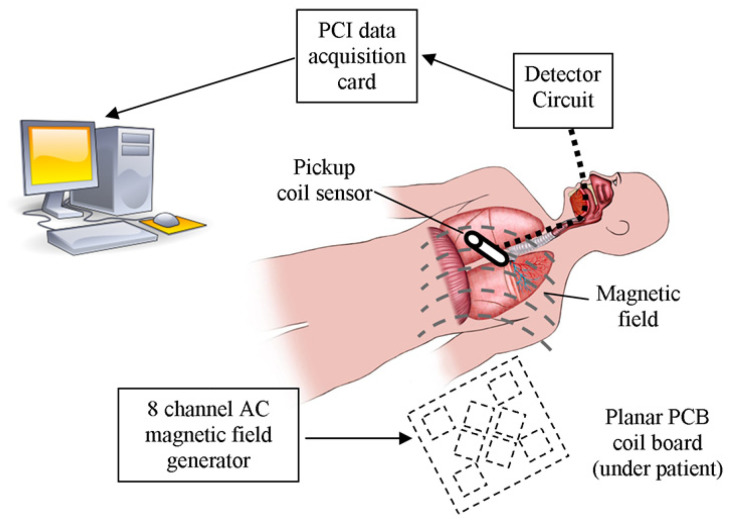
An overview of the entire system. A set of magnetic coils placed below a patient generate an AC magnetic field which is detected by a sensor which in turn is processed to determine an estimate of the sensor’s position [17].

Figure 3 shows an overview of the entire system. A set of magnetic coils placed below a patient generates an AC magnetic field which is detected by a sensor. The sensor voltage is in turn processed to determine an estimate of the sensor’s position. The magnetic field generator (FG) is often planar in its conduction to allow it to be easily positioned below the patient on the operating table. A data acquisition unit is used to digitize the induced sensor voltage and the measured voltage is then converted to a position and orientation using (3).

### 2.2. Radiolucent Materials

The field generator array is typically created from copper coils [17,22,23,24]. Many conventional coils contain X-ray attenuating materials that are visible in the X-ray images and potentially obscure patient anatomy. The linear attenuation coefficient of dense materials varies approximately as the fourth power of the atomic number for X-ray energies in the diagnostic range [25]. Aluminum (Al), with an atomic number of 13, has, therefore, considerably less X-ray attenuation than copper, with an atomic number of 29. However, copper is a better conductor and easier to work with generally [26].

In order to predict how easily an X-ray will penetrate a material, its attenuation coefficient needs to be considered. The attenuation coefficient characterizes how easily a volume of material can be penetrated by an X-ray or other ionizing energy. For all materials, this is a highly non-linear parameter based on a wide range of photon scattering and absorption processes [25]. However, tables of the attenuation coefficient are readily available [27], which allows comparisons of the relative absorptions of different materials to be analyzed. To predict the absorption of a monochromatic X-ray beam as it traverses a homogenous material with an absorption coefficient *µ*, the Lambert–Beer law can be used

(4) $$I=I_{0}e^{-\mu x}$$

where the observed intensity *I* is related to the intersection length of the object *x* and $I_{0}$ is the X-ray intensity at its source [28]. If we define the normalized intensity of the X-ray source with distance into a material as:

(5) $$\alpha =\frac{I}{I_{0}}=e^{-\mu x}$$

In order to compare the absorption of two materials, we define a simple ratio of the change in absorption relative to the ideal case where no attenuating materials are present based on the relative attenuation of two materials denoted *A* and *B* as follows:

(6) $$\gamma_{AB}=\frac{1-\alpha_{A}}{1-\alpha_{B}}$$

**Figure 4:**
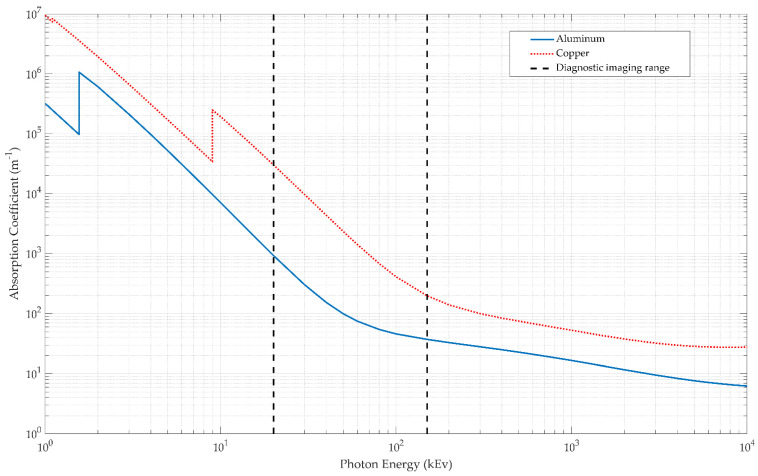
A comparison of the absorption coefficient for pure copper and aluminum for a range of photon energies. The typically used range for diagnostic imaging is also shown. Within this range, we see that copper has a much greater absorption than aluminum and this trend is more pronounced at lower energy levels [29,30].

**Figure 5:**
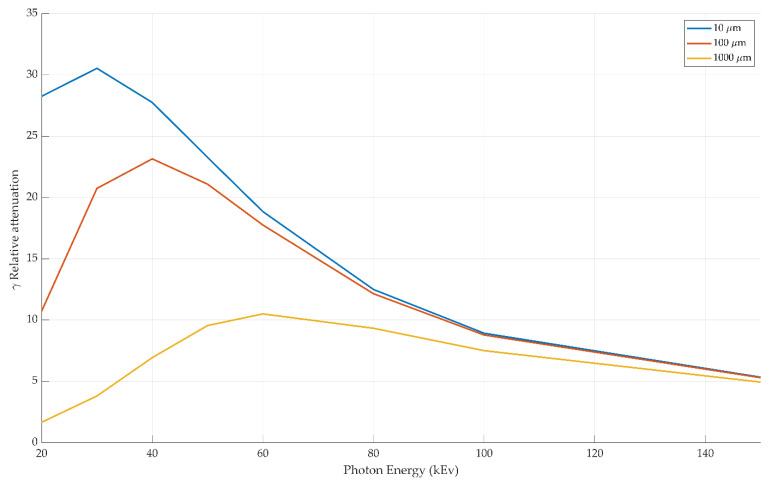
A comparison between the relative absorption over copper to aluminum for a range of material thicknesses. At low voltages, the absorption varies significantly at the different material thicknesses but these differences decrease as the voltage increases across the typical diagnostic imaging range [29,30].

For example, for a 50 μm thick layer of aluminum and copper exposed to an X-ray tube voltage of 80 keV would result in γ=12.338, i.e., the copper is approximately 12 times more attenuating in comparison to the aluminum. The voltages used in diagnostic X-ray tubes range from roughly 20 kV to 150 kV and thus the highest energies of the X-ray photons range from roughly 20 keV to 150 keV [25]. An example of this can be seen in Figure 4. Figure 5 shows a comparison between the relative absorption of copper to aluminum for a range of material thicknesses. At low voltages, the absorption varies significantly at the different material thicknesses but these differences decrease as the voltage increases across the typical diagnostic imaging range.

### 2.3. Transmitter Circuit

**Figure 6:**
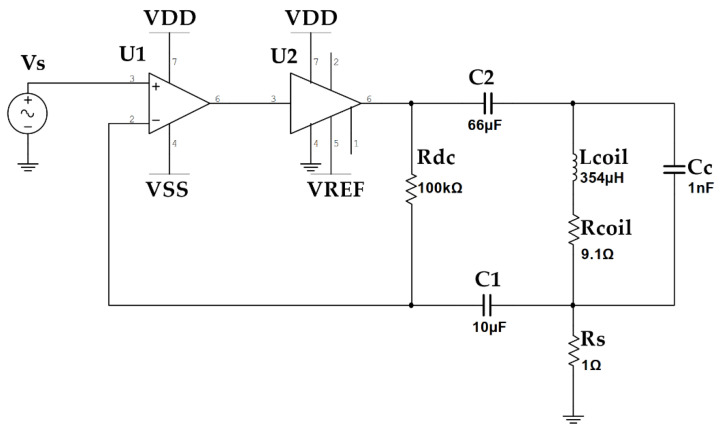
The power amplifier circuit used to deliver sinusoidal AC current to each of the eight coils used in the field generator coil array. *U*_1_ is the LM7171 high speed operational amplifier and *U*_2_ is an LMH6321 current buffer. *C*_2_ blocks any DC current from the coil, *C_c_* reduces the AC impedance of the coil at higher frequencies to improve the stability of the circuit. A precision current sense resistor is used to feedback the measured current.

A modification of the circuit is described in our earlier work [17]. An LMH6321 current buffer is used in a constant AC-current configuration to deliver the drive current to each coil. The circuit used is shown in Figure 6. A single 15 V supply is used to power each buffer. The signal is sourced from an on-board programmable sine wave generator. At 15 V input, the LMH6321 can provide a maximum undistorted output voltage swing of 4.1 Vrms, with a maximum current of 300 mA. These drive limits are used in the optimization process for the coil design. To reduce heat generation, the output peak current of each channel is limited to 150 mA.

*U*_1_ is the LM7171 high speed operational amplifier and *U*_2_ is an LMH6321 current buffer. *C*_2_ blocks any DC current from the coil, *C_c_* is there to reduce the AC impedance of the coil at higher frequencies to improve the stability of the circuit. A precision current sense resistor is used to feedback the measured current. The transmit frequency of each coil was configured as 2020, 2240, 2580, 2820, 3020, 3320, 3640, and 4000 Hz. The spacing of the frequencies was chosen to minimize intermodulation products in both the transmit and receive amplifier stages.

The coils used for the aluminum field generator were measured to have a DC resistance of 9.1 Ω and an inductance of 354 µH. At the maximum operating frequency of 4 kHz, this results in an impedance of 12.7 Ω as given by (7) [31]. At a drive current of 150 mA peak, this results in a peak voltage of 1.9 V. This is well within the range of the LMH6321 linear range and low distortion range.

(7) $$\left|Z_{\max}\right|=\sqrt{R^{2}+{\left(2\pi fL\right)}^{2}}$$

### 2.4. Planar Printed Circuit Board (PCB) Coils

To generate the magnetic fields necessary for this system, planar windings on a printed circuit board were utilized. This is a low cost and precise method for creating accurate magnetic fields and their associated models for tracking applications [17,32,33]. Square coils were chosen as they simplify the required calculations necessary in comparison to circular and other possible coil formations. If we consider that the coil is formed from a number of straight filaments, by calculating the magnetic field due to each filament as per the Biot–Savart law [17,33,34], the magnetic field can be accurately calculated. This is explored in more detail in our earlier work [17].

Along with calculating the magnetic field distribution for each coil size, the inductance and resistance were also calculated to ensure that they were below the specified limits. The resistance was simply calculated by determining the total length of the coil and using (8) where ***ρ*** is the resistivity of copper (1.68 × 10^−8^ Ωm) [31].

(8) $$R=\rho \frac{l_{coil}}{wt}$$

The inductance was calculated using the modified Wheeler formula which is given by (9), where *K*_1_ and *K*_2_ are shape-dependent parameters, which for a square coil have values of 2.34 and 2.75, respectively [35], and *µ*_0_ is the permeability of free space. The two parameters $l_{avg}$ and χ are dependent on the distribution of the coil and are given by (10) and (11), where $l_{out}$ is the outer side-length of the coil and $l_{in}$ is the inner side-length of the coil.

(9) $$L=K_{1}\mu_{0}\frac{N^{2}l_{avg}}{1+K_{2}\chi }\;$$

(10) $$l_{avg}=\frac{l_{out}+l_{in}}{2}$$

(11) $$\chi =\frac{l_{out}\;-l_{in}}{l_{out}+l_{in}}$$

The relative permeability also has an effect as it affects the skin depth of the material. The skin depth for conductive media in meters is given by:

(12) $$\delta =\sqrt{\frac{2\rho }{\omega \mu_{0}\mu_{r}}}$$

where ***ρ*** is the resistivity of the material in units of Ωm, ω is the angular frequency in rad/s, and *µ_r_* is the relative permeability of the material. For the coil dimensions and the frequency of operation for these coils, the skin depth can be neglected.

**Figure 7:**
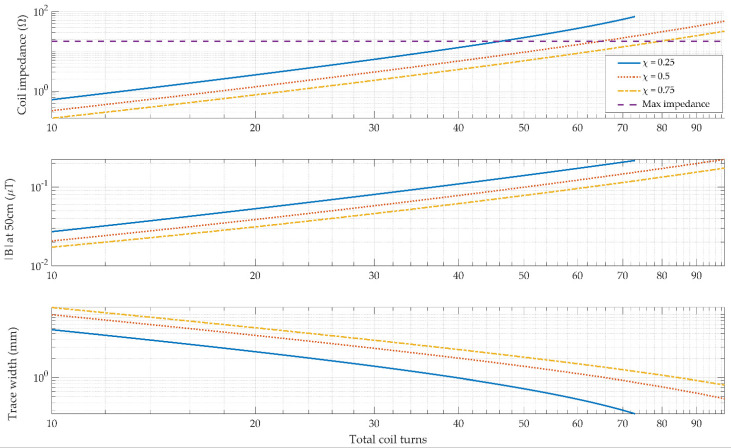
A parametric analysis of the effect of increasing the number of turns in the coil for a range of different packing factors. It was observed that at very large and very small values of the packing factor, the generated magnetic field tended to reduce.

Looking at the three packing factors analyzed, the peak magnetic field at a distance of 50 cm from the coils were 130.6 nT, 134 nT, and 131.5 nT at a packing factor of 0.25, 0.5, and 0.75, respectively as shown in Figure 7. It was observed that at extreme values of the packing factor (i.e., outside the range 0.1 < χ < 0.9), the generated magnetic field tended to reduce. A packing factor of approximately 0.5 generally gives the best performance overall.

Exposure to alternating magnetic fields by users is a key consideration in the design field generators for electromagnetic tracking. The International Commission on Non-Ionizing Radiation Protection (ICNIRP) provides guidelines with regard to the maximum magnetic field exposure at a particular frequency [36]. In our operating range of 2 kHz to 4 kHz, the maximum occupational exposure is limited to 100 μT RMS [36]. In the case where multiple simultaneous frequencies are generated as is the case in this design, the magnitudes of the all carrier frequencies must be combined to see the cumulative effect of the exposure. Since there are 8 coils transmitting simultaneously, the actual field strength per coil must be approximately divided by 8 to ensure that the total contribution of each coil does not exceed the limits.

**Figure 8:**
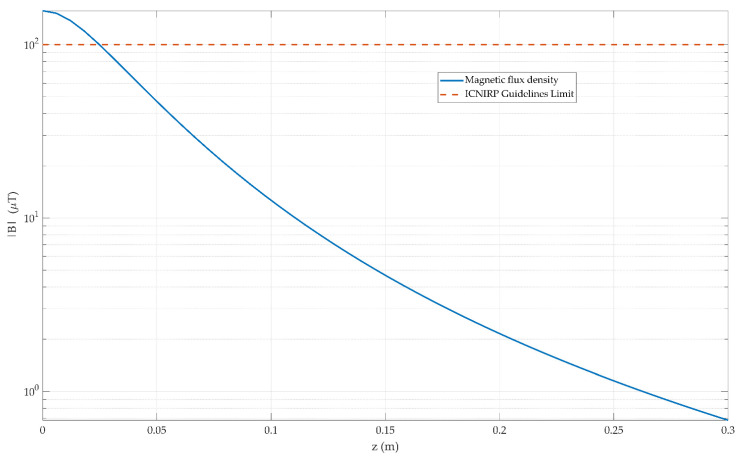
The predicted on-axis magnetic field of the aluminum coils carrying a peak current of 150 mA is shown in this figure. The ICNIRP guidelines limit for the maximum operating frequency of 4 kHz is shown, which is 100 μT for occupational use.

The predicted on-axis magnetic field of the aluminum coils carrying a peak current of 150 mA is shown in Figure 8. The ICNIRP guidelines limit for the maximum operating frequency of 4 kHz is shown, which is 100 μT for occupational use. Close to the coil this guideline is exceeded but this is within the confines of the enclosure used. The contribution of each coil transmitting together also affects the net magnetic field; however, the field roll-off from the coils is very abrupt as can be seen in the figure and the other coil contribution can be neglected.

### 2.5. Sensors

**Figure 9:**
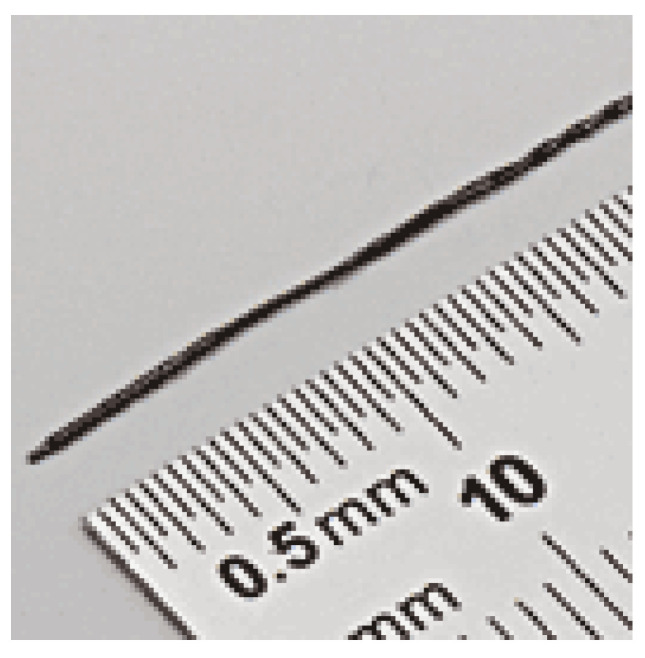
An example of the sensors used in this experiment, an Aurora 5-DOF sensor by NDI.

Commercially available sensors by NDI (Waterloo, ON, Canada) were used with our system. They were chosen as they have a high sensitivity and are available in small sizes allowing for easy integration in custom medical devices. To fully understand the operation of the NDI sensors, our prior work carried out an extensive range of tests to evaluate their sensitivity, frequency response, and linearity [37]. These tests were carried out with an unshielded 5-DOF sensor. These sensors are low cost and readily available and only a handful of other suitable sensors are available on the market as off-the-shelf components. An example of this sensor can be seen in Figure 9.

### 2.6. Sensor Interface

An INA163 instrumentation amplifier was used to amplify the small signal generated by the sensor coils. This voltage was then digitized using an NI-6212 Data Acquisition Unit sampling at 100 kHz. This recorded signal was demodulated and the position and orientation of the sensor were determined using the methods described in our earlier works [17].

### 2.7. Aluminium Coils

Advanced PCB design techniques exist for creating printed circuit board traces made from aluminum, which has a very low density and has substantially lower X-ray absorption than other commonly used conductors. This would be a minor modification of our pre-existing planar PCB designs. Our previous work utilized coils that have a two-layer construction of 70 μm thick copper [17,38]. New aluminum coils have a similar two-layer construction but with 50 μm thick foil. Our recent higher power coil designs use six-layer PCBs with 105 μm copper to keep the resistance low.

**Figure 10:**
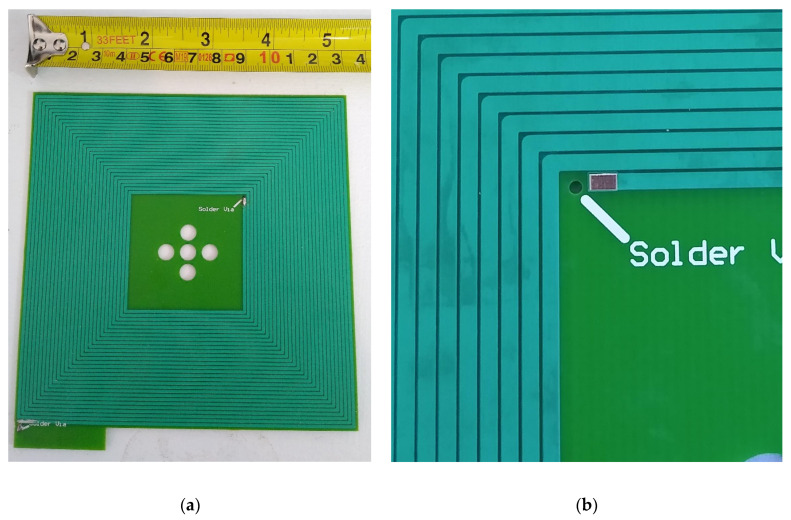
(**a**) The two-layer aluminum coil used in these experiments. (**b**) A close-up of the jumper wire slot required in place of traditional copper vias.

There are a number of issues inherent in the PCB design methodology of aluminum coils including solderability being much reduced and layer-to-layer vias not being possible using standard methods, increased coil resistance which limits maximum field strength, and generally being limited to two-layer construction. Standard PCB vias are not possible with the aluminum-only process that was chosen for this development. In place of standard vias, small jumper wires were required to bridge the aluminum conductors from the top to bottom layer of the PCBs. This is shown in Figure 10.

### 2.8. Coil Arrays and Enclosure Design

**Figure 11:**
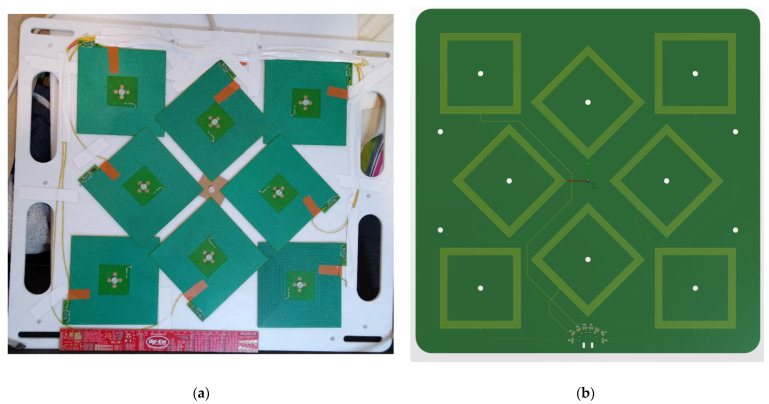
(**a**) The assembled aluminum coil array in its Ertalyte enclosure. There is some overlap of the coils due to the constraints of the enclosure dimensions. The connecting wires were arranged on the outside of the coils to minimize their interference with the X-ray imaging. (**b**) The copper field generator array used in our standard EMTS, the coils were arranged on a single PCB to allow precise positioning of each coil relative to one another.

**Figure 12:**
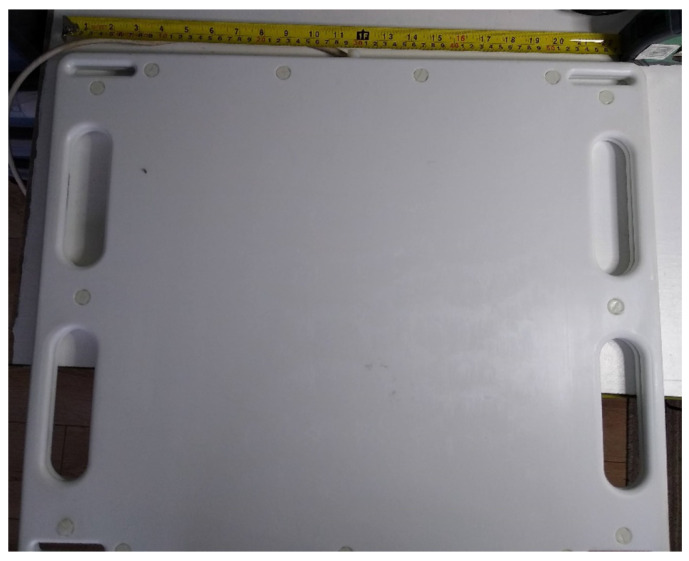
The assembled aluminum coil array in its Ertalyte enclosure.

The coil arrays are housed in a machined thermoplastic enclosure formed out of Ertalyte, a type of polyester. The two faces of the flat enclosure were machined from 10 mm sheets of the material shown in Figure 11 and Figure 12. For the aluminum coils, precise mountings holes were machined from the plastic to ensure the coils were correctly located. For the copper field generator, all the coils were created on a single FR4 PCB to allow precise positioning of each coil relative to one another. Substrates this large were not possible with the aluminum coil manufacturing process.

### 2.9. X-ray Imaging Testing

To evaluate the X-ray absorption of the aluminum coils in comparison to other similar copper coils, two experiments were performed. The first experiment utilized an industrial X-ray imaging device typically used for failure analysis to obtain high resolution images of the coils. A second experiment used a medical grade CT imager to image the coils; this was to give a more representative exploration of a more typical use case.

For the first test, a Phoenix VTOMEX L 300 microCT imager from Waygate Technologies was used. The device was configured with an excitation voltage of 120 kV, a tube current of 100 μA, and a pixel size of 135 μm with 20 image integrations.

**Figure 13:**
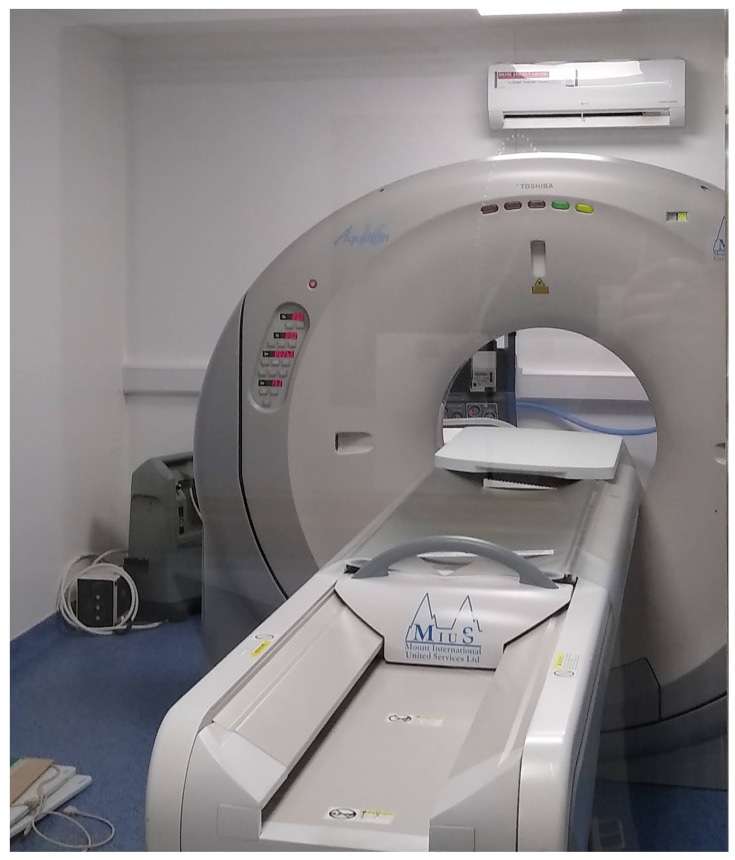
A traditional medical Toshiba Aquilion CT was used to image the coil configurations.

For the medical use case experiment, a Toshiba Aquilion CT was used. The device was configured with a 120 kV voltage, with 100 mAs tube current, and a slice thickness of 4 mm. The resulting voxel size of this device was 4 mm in comparison to 135 μm for the microCT imager. As a result, some of the quantitative data derived from the images is not as reliable. The Toshiba Aquilion CT used can be seen in Figure 13.

Both systems used create images in different modes. The Phoenix VTOMEX L 300 can create full CT 3D images or standard 2D X-ray images. The Toshiba Aquilion is generally limited to reconstructed CT images for certain orientations, but 2D X-ray images are possible when used in the scout mode, typically used for aligning the imager with a low dosage. CT image sets are generally slices of images and not a direct X-ray image, which is the resulting absorption projection of the materials through the entire material. Data presented in this paper from the Phoenix VTOMEX L 300 are all 2D, planar X-ray data whereas the data presented from the Toshiba Aquilion are a mixture of CT rendered images and 2D scout image X-rays. This is noted on each dataset where necessary.

**Figure 14:**
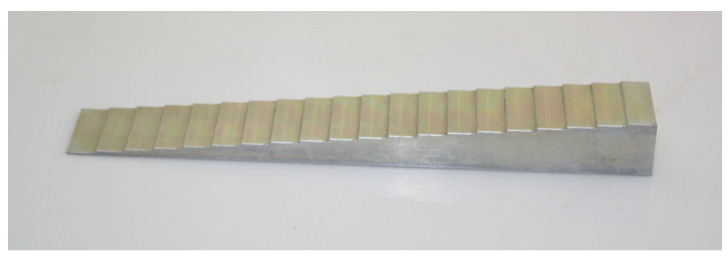
A pure aluminum step wedge was used as an X-ray absorption reference. This is a staircase profile aluminum block that increments in steps of 1 mm from 1–20 mm and is frequently used in the calibration and testing of X-ray systems.

As a reference to the X-ray absorption of the materials in the acquired images, an aluminum step wedge was used. This is a staircase profile aluminum block that increments in steps of 1 mm from 1–20 mm and is frequently used in the calibration and testing of X-ray systems [39]. The step wedge was used to give a reproducible baseline to the results shown in this work. This tool can be seen in Figure 14.

## 3. Results

### 3.1. Position Accuracy

As with any navigation system, the key performance metric is the accuracy of the reported position data. The accuracy performance of the new coil array was analyzed using an identical method as described in our earlier work [17] where Lego Duplo blocks are used to accurately position the sensor in known positions and increments. The sensor was positioned at a height of 20 cm above the field generator and a planar grid of 49 test points was obtained. This was compared against results obtained with our standard copper coil generator. Equation (3) was solved using Mathworks MATLAB where the non-linear least squares function *lsqnonlin* was used to minimize the error between the measured flux by the sensor and the magnetic field model of the system resulting in the position of the sensor.

The position error between the real sensor location $\left(x_{r},y_{r},z_{r}\right)$, and the calculated position, $\left(x_{c},y_{c},z_{c}\right)$, was determined at each test point using (13).

(13) $$e_{p}=\sqrt{{\left(x_{c}-x_{r}\right)}^{2}+{\left(y_{c}-y_{r}\right)}^{2}+{\left(z_{c}-z_{r}\right)}^{2}}$$

The distance from the center of the coil array is calculated with (14).

(14) $$r=\sqrt{x_{r}{}^{2}+y_{r}{}^{2}+z_{r}{}^{2}}$$

All the position results that were calculated were rounded to the nearest 100 µm.

**Figure 15:**
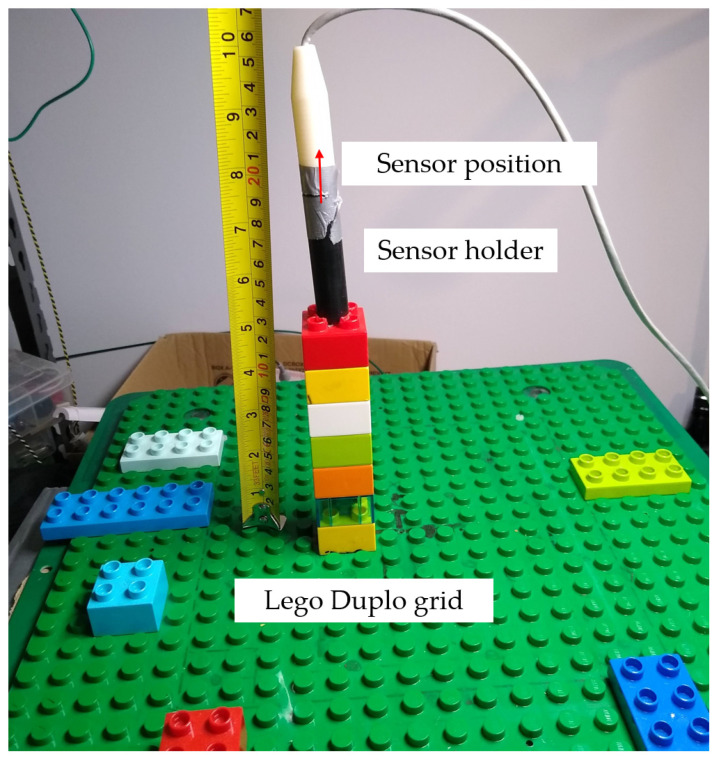
The test setup used for accuracy testing. The sensor was positioned at a height of 20 cm above the field generator and a planar grid of 49 test points was obtained. For easy positioning of the tiny sensors used with the system, a high tolerance sensor holder was machined to hold the sensor in place in the Lego Duplo blocks.

**Figure 16:**
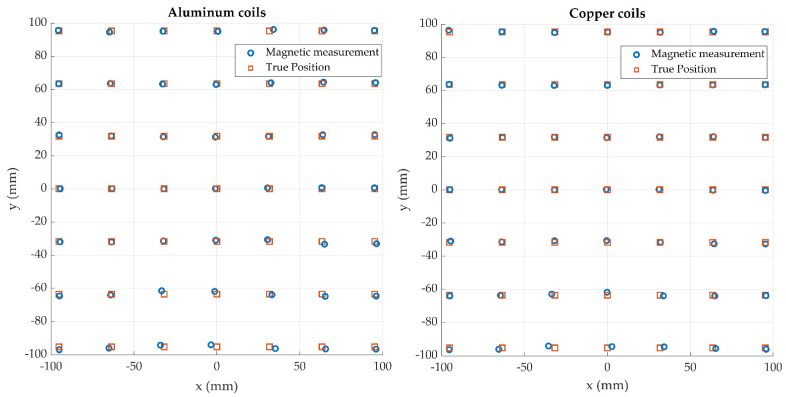
Sensor position results for both sets of coils with the sensor was positioned at a vertical height of 20 cm. The true sensor position as calculated from the Lego Duplo block positions is displayed alongside its calculated position determined by solving Equation (3).

**Figure 17:**
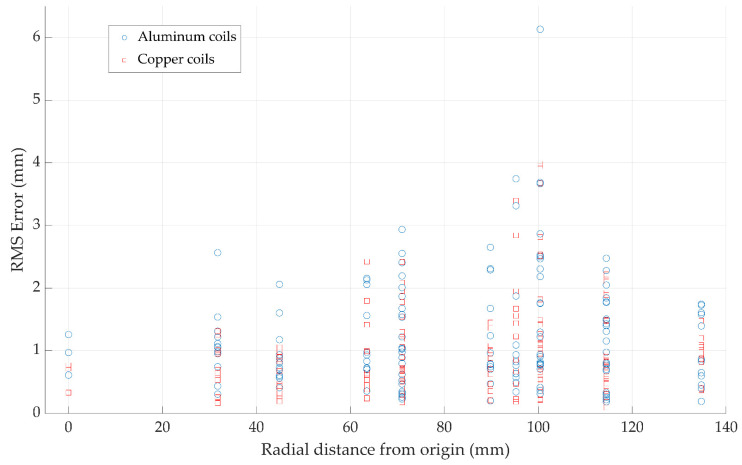
Position results from three repeated tests are plotted here. The distance from the center of the field generator to each test point is plotted to give a representation of the spatial spread of the data.

For easy positioning of the tiny sensors used with the system, a high tolerance sensor holder was machined to hold the sensor in place in the Lego Duplo blocks. This is shown in Figure 15; the approximate sensor position is also shown in the figure. The mean, RMS, standard deviation, and 90th percentile of the position error of each test point is calculated and shown in Table 1. Each dataset was gathered three times to give clearer statistical insights. The results of the experiment are shown in Figure 16 and Figure 17.

### 3.2. Radiolucency Analysis

An investigation into the X-ray absorption of the aluminum coils was performed. A step wedge was used as an absorption reference. The wedge provided 1 mm increments of aluminum and provides a baseline with which to compare the absorption levels of materials. Three coil formations were analyzed:

- Two-layer, 70 μm thick copper coil
- Six-layer, 105 μm copper coil
- Two-layer, 50 μm aluminum coil

**Figure 18:**
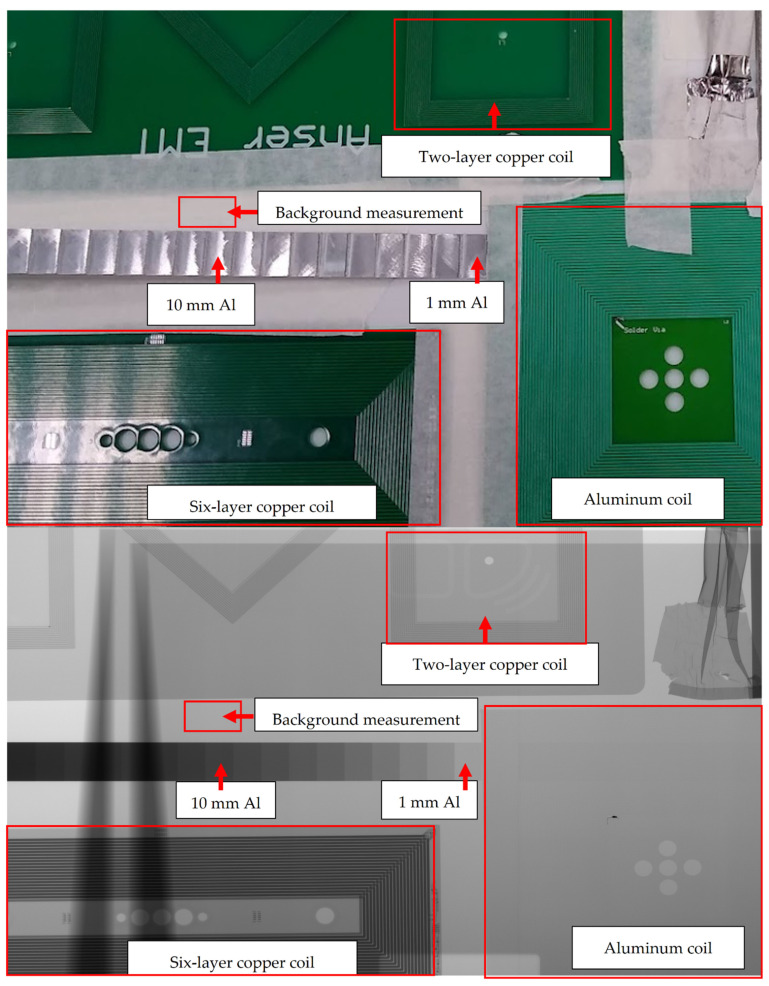
X-ray analysis of the 3 different coil designs with a 1–20 mm aluminum step wedge for reference.

**Figure 19:**
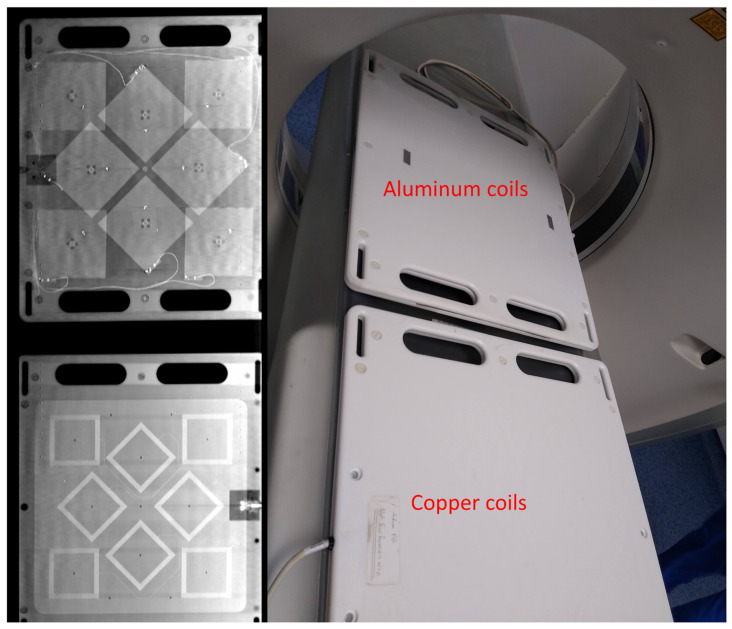
A direct comparison of the two different field generator designs. Both devices were put through the CT scanner together to allow for easier comparisons. From the aluminum coils, the FR4 substrate is visible but the actual coils cannot be discerned. This is in stark contrast to the copper coils which can be clearly identified.

**Figure 20:**
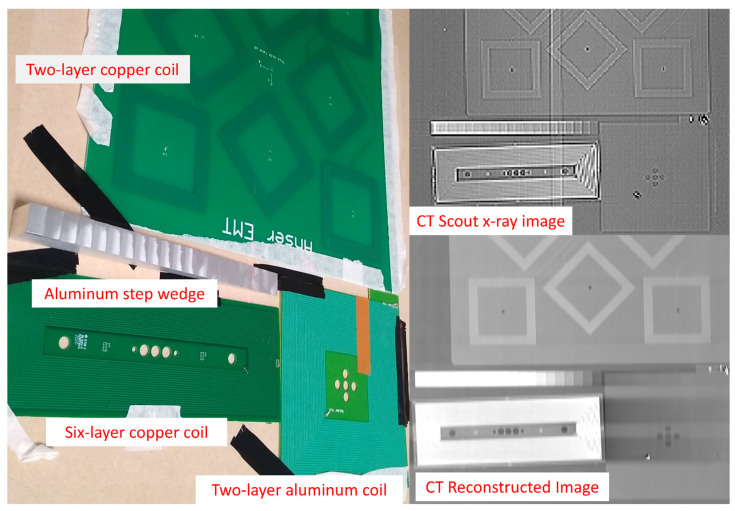
A similar assembly of test coils were scanned using the Toshiba Aquilion scanner. On the left of this image, the combination of the 2-layer aluminium coils, 2-layer copper coils, and a 6-layer coil design are positioned along with the aluminum step wedge. On the top right, we see the X-ray image from the CT scout X-ray image and bottom right we see an image resulting from the average, a 20 mm stack of reconstructed images.

Figure 18, Figure 19 and Figure 20 show the test setups and images used in the analysis of the X-ray absorption of the coils under test.

**Figure 21:**
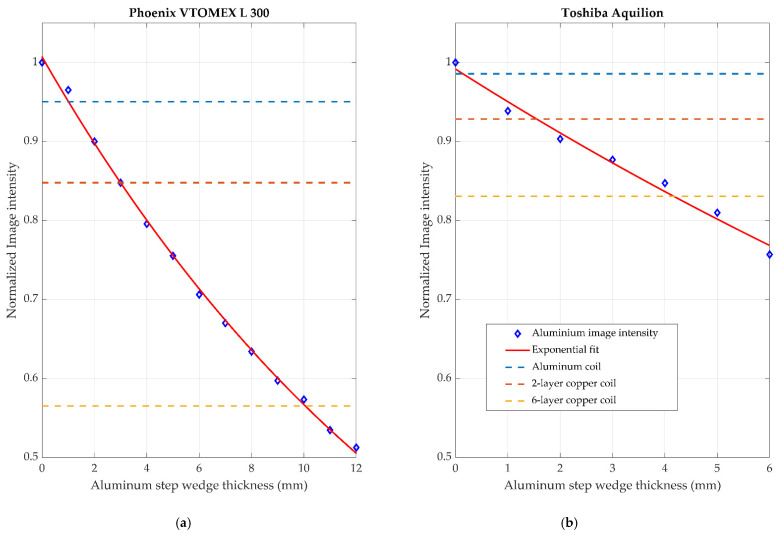
The normalized intensity of the image background in reference to the aluminum step wedge thickness. (**a**) shows the results from the Phoenix VTOMEX L 300 industrial microCT scanner and (**b**) shows the results from the Toshiba Aquilion medical CT scanner. The intensity of each of the materials is normalized to the measured background intensity of the X-ray images used.

Figure 21 shows the normalized intensity of the image background in reference to the aluminum step wedge thickness. In this figure, the intensity of each of the materials was normalized to the measured background intensity of the X-ray images used. The background intensity was determined by taking the average intensity of the background area of the image shown in Figure 18. This region was chosen as it was known to contain minimal radiopaque materials and had approximately the lowest intensity across the entire image. This background intensity value was then used to normalize all measurements presented in this analysis. This normalization technique was useful to determine the relative absorption of the materials under test. The image data from the Toshiba Aquilion were inverted so they matched the format of the Phoenix VTOMEX L 300. The data were inverted by subtracting the unsigned 8-bit image data from 128, which is the maximum intensity possible in this data type. An exponential fit as suggested by the Lambert–Beer law was also applied to the figure. The relative absorption of the materials is shown in Table 2. The data between these two systems are similar in appearance but the underlying operation of each system makes a direct comparison irrelevant.

## 4. Discussion

### 4.1. Position Accuracy

From the accuracy metrics shown in Section 3.1, it is clear that both field generator assemblies provide similar levels of accuracy. This was an expected result since the change from copper to aluminum in the coil design has little overall effect on the underlying physics of the system. The aluminum coils bring with it a host of practical engineering challenges. Among the limitations of the aluminum coil manufacturing process was the maximum PCB dimensions of approximately 20 cm × 30 cm, which is significantly less than the 42 cm square dimensions of our other designs. This resulted in the use of multiple smaller boards instead of a single large PCB carrying all the coils. The effect of this is that the overall precision of the coil locations and its associated magnetic field models decreased, and this resulted in the slight decrease in accuracy of the sensor positions. With optimization of the manufacturing processes of the aluminum coils, larger coil arrays are possible, and this will mitigate any of these larger error effects of this prototype.

A further limitation of the aluminum coils in comparison to copper is the increased resistivity of the material. For this small tracking volume, it is not a significant problem; however, for larger electromagnetic tracking applications, this could become a limiting factor due to excessive heat generation in the coils when higher current densities are used.

### 4.2. Radiolucency

The reduction in the visible footprint of the coils through the use of aluminum from these tests was clearly demonstrated. In most cases, the absorption of the FR4 base material that supports the coils was a larger contributor than the coils themselves in the case of the aluminum coils. In both imaging examples demonstrated, at least a 60% reduction in the absorption of the X-ray source was observed by the two-layer aluminum coils compared to the two-layer copper coil relative to the background image intensity. While some of these results may seem trivial, reducing these kinds of image artifacts can be critical in certain applications, such as the imaging of pulmonary arteries, for example, where artifacts could potentially be mistaken for other medical devices such as stents or guide wires.

For both imaging systems used, the overall trend was similar. Of all the coil formations analyzed, the aluminum coils had the lowest absorption and, as expected, the thicker layers of copper seen in the six-layer example had the largest impact on the images. Both systems were operated at the same imaging voltage of 120 kV. However, as can be seen in Figure 21, the resulting normalized intensity is significantly different. This is due to the fact that there are many differences between the two systems; their end applications are radically different, so the image processing on the data and the degree of image averaging is different. The achieved voxel size of the medical CT system was also significantly less than the microCT data, which resulted in more reliable and consistent data.

## 5. Conclusions

The design and construction of an aluminum coil array for electromagnetic tracking applications has been presented. The accuracy of this new field generator has been shown to be 1.5 mm RMS, which was only marginally worse in comparison to the standard coil arrays used with our system. From a comparison of two different X-ray imaging modalities, the low X-ray absorption of the aluminum coils was clearly demonstrated. The creation of effectively radiolucent electromagnetic tracking systems allows the usage of the technology to be applied to a wide range of different procedures where interference with the imaging modalities used is a major concern.

## Figures and Tables

**Table 1:** Position accuracy results for the two different coil formations tested. The mean, RMS, standard deviation, and 90th percentile of the position error of each test point is calculated and shown. Each dataset was gathered three times to give clearer statistical insights.

Field Generator	Mean Error (mm)	RMS Error (mm)	Standard Deviation Error (mm)	90th Percentile Error (mm)
Standard copper	0.99	1.51	0.76	2.39
Aluminum	1.23	1.25	0.88	1.95

**Table 2:** The normalized intensity of the image background in reference to the aluminum step wedge thickness. The intensity of each of the materials was normalized to the measured background intensity of the X-ray images used.

Material	Phoenix VTOMEX L 300	Toshiba Aquilion
Two-layer aluminum	0.95	0.98
Two-layer copper	0.85	0.93
Six-layer copper	0.57	0.83

## Data Availability

Data and code used in this paper can be found at https://github.com/kilianod5150/Radiolucent_Coils (accessed on 11 May 2021).
